# PATTERN: Pain Assessment for paTients who can’t TEll using Restricted Boltzmann machiNe

**DOI:** 10.1186/s12911-016-0317-0

**Published:** 2016-07-25

**Authors:** Lei Yang, Shuang Wang, Xiaoqian Jiang, Samuel Cheng, Hyeon-Eui Kim

**Affiliations:** 1 Department of Electrical and Computer Engineering, University of Oklahoma, Tulsa, OK 74135 USA; 2 Department of Biomedical Informatics, University of California, San Diego, 92093 USA

## Abstract

**Background:**

Accurately assessing pain for those who cannot make self-report of pain, such as minimally responsive or severely brain-injured patients, is challenging. In this paper, we attempted to address this challenge by answering the following questions: (1) if the pain has dependency structures in electronic signals and if so, (2) how to apply this pattern in predicting the state of pain. To this end, we have been investigating and comparing the performance of several machine learning techniques.

**Methods:**

We first adopted different strategies, in which the collected original n-dimensional numerical data were converted into binary data. Pain states are represented in binary format and bound with above binary features to construct (n + 1) -dimensional data. We then modeled the joint distribution over all variables in this data using the Restricted Boltzmann Machine (RBM).

**Results:**

Seventy-eight pain data items were collected. Four individuals with the number of recorded labels larger than 1000 were used in the experiment. Number of avaliable data items for the four patients varied from 22 to 28. Discriminant RBM achieved better accuracy in all four experiments.

**Conclusion:**

The experimental results show that RBM models the distribution of our binary pain data well. We showed that discriminant RBM can be used in a classification task, and the initial result is advantageous over other classifiers such as support vector machine (SVM) using PCA representation and the LDA discriminant method.

## Introduction

Pain is very important in patient care, and more than half of hospitalized patients have reported pain [1]. In America, chronic pain affects about 100 million people [2]. Pain and its associated problems are among the leading public health problems in the US [3]. Although pain assessment guidelines are available, pain management is still deemed insufficient as reported by many patients and health professionals [4]. Pain management relies on the ability to accurately assess when, how and to what extent a patient is experiencing pain. As a subjective phenomenon, the severity of the perceived pain may vary significantly among different patients. Thus a patient’s self-report is usually treated as the most reliable pain measurement [5]. Various non-physiological factors such as emotional state, environmental and socioeconomic contexts [6–8], etc. may also have an impact on pain assessment, which makes accurate assessment of pain a non-trivial task.

The multidimensional pain theory [9] proposed by Melzack categorizes pain based on non-observable (i.e., subjective), and observable (i.e., objective) indicators. For example, patient’s self-reports of pain that include sensory, emotional, and cognitive components of pain are subjective information, which can serve as non-observable indicators. Nurses often assess and document this information. Observable indicators include the physiological and behavioral components of pain, which are usually captured and documented in critical care settings through continuous monitoring. The behavioral components are actively applied in behavioral observational pain scales. The physiological signals should also help healthcare professionals better perform the pain assessment. Although many studies have attempted to associate physiologic signals and pain [10–20], few practical and reliable methods of using physiological components in pain assessment are available. In this study, we attempted to assess the probability of pain presence based on physiological data. This approach can particularly be useful for caring minimally responsive patients who cannot make self-report of pain.

A great deal of effort has been made to analyze pain. Recently, there has been increasing interest in exploring the task of predicting pain state using machine learning techniques. Generally, the task of prediction requires discovering (learning) patterns in training data. A good data model to represent the distribution of training data is critical in this process. Our previous work [21] was done using the electronic flowsheet data of ICU patients collected for a limited time interval. These time series data were projected into a lower-dimensional subspace, and a number of data vectors (within a time window size) were represented (reconstructed) with some linear combinations of principal components (PCs). The magnitude of residual between original and reconstructed data can be used to measure the level of pain. In this previous study, we did not utilize any label (i.e., documented pain presence) from the data. It is a good strategy to deal with the time series data when the recorded labels are incomplete. On the other hand, ignoring all the labels may result in loss of significant information.

The overall approach of the study reported here is different from that of the previous one in that: (1) we focus on learning from labeled data, (2) we treat our data as non-temporal rather than temporal data, and (3) we focus on investigating the relationships between activation of pain and the normal/abnormal state of various electronic signals. Consequently, the problem is transformed into a supervised learning and classification problem.

In machine learning, linear methods [22] are fast and robust which successfully avoid the over-fitting problem. In addition, they are guaranteed to produce a global optimum. However, they are often too limited when used in real world data. In this study, we employ another alternative non-linear model, restricted Boltzmann machine [23] (RBM), a deep learning approach. In this study, we trained RBM with the labeled data and feature vectors in a supervised manner. Both the feature and the labels are visible units in the model. Moreover, using the nature of this model, discriminant RBM can be used in the classification. The probability of unknown label class can be calculated through free energy when a new input was fed into RBM. In the rest of this paper, we will present the proposed framework of our new pain prediction algorithm called PATTERN: Pain Assessment for paTients who can’t TEll using Restricted Boltzmann machiNe.

## Background

### Data representation

The success of a classification algorithm highly depends on the choice of representation for data. One hypothesis is that different representations can more or less entangle and hide the different explanatory factors of variation behind the data [24, 25]. An attractive alternative is to estimate the parametric distribution, which explains the data best, for example, Gaussian Model and Gaussian Mixture Models. Another concern is the high data dimensionality. Higher dimensional data can provide richer and detailed information than lower dimensional one; at the same time, learning from the high dimensional data often suffer from over-fitting problem. In other words, with an insufficient number of data points in the training set, we tend to memorize each data point rather than learn from it. To avoid over-fitting, some classical approaches typically project the data into lower-dimensional space, such as principal components analysis (PCA). Recently, using RBMs to model the data have been reported in a large variety of learning problems [26–28]. Theoretically, the capacity of RBMs has been demonstrated that it can provide a powerful means to representing data [29].

### Restricted Boltzmann machine (RBM)

A RBM is an undirected probabilistic graphical model with symmetric connections between hidden (latent and usually is binary-valued) and visible (observed) variables to model an input distribution. Unlike most linear models that try to perform transformation in the same space with input data, RBM introduced a new type of unobserved variables, which increase the representative power of the model. The word “restricted” suggests there is no connection between the units from the same layer, this restriction makes learning much easier than Boltzmann machine. Precisely, let ***v*** = {*v*_*1,*_*v*_*2,*_*…,v*_*m*_} represents a visible vector and ***h*** = {*h*_1_, *h*_2_, …, *h*_*n*_} denotes a hidden vector.

**Fig. 1:**
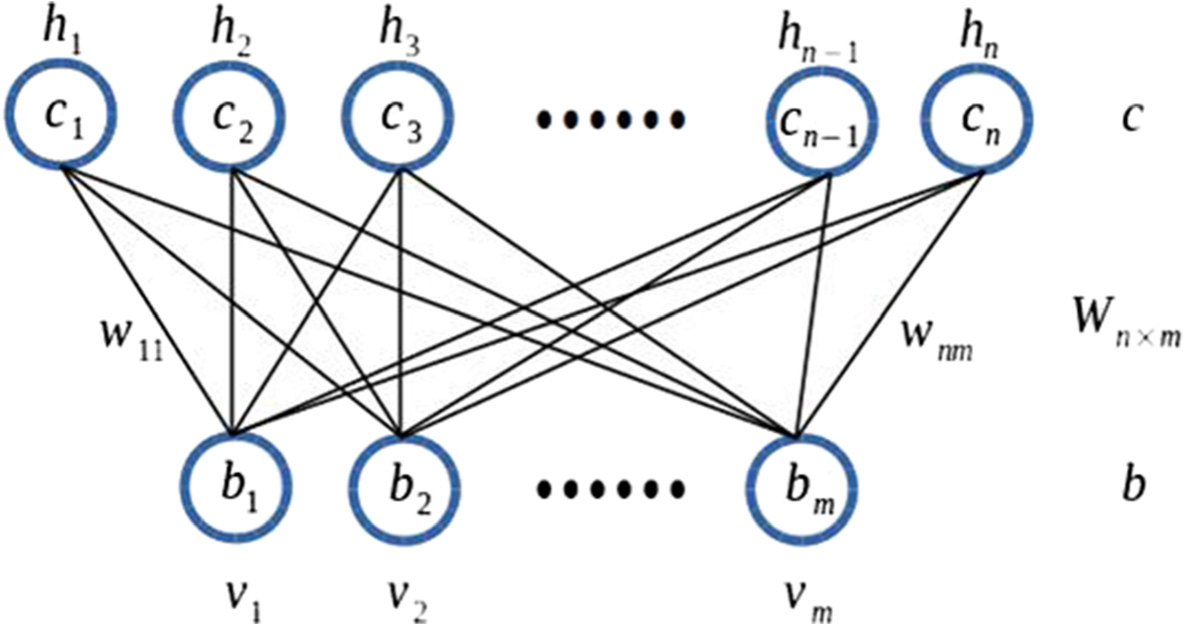
The Graphical Model of Representation for an RBM

An RBM (Fig. 1) defines a distribution over ***v*** and ***h*** through energy function:

1 $$ E\left(\boldsymbol{v},\ \boldsymbol{h}\right)=-{\displaystyle {\sum}_i{b}_i{v}_i-{\displaystyle {\sum}_j{c}_j{h}_j-{\displaystyle {\sum}_{i,j}{v}_i{h}_j{w}_{ij}}}} $$

Where *v*_*i*_, *h*_*j*_ ∈ {0, 1} are the binary values of visible variable *v*_*i*_ and hidden variable *h*_*j*_, and *b*_*i*_, *c*_*j*_ are the biases from visible and hidden layers, respectively. *w*_*ij*_ is the weight parameter between *v*_*i*_ *and h*_*j*_. We assigned a probability to each pair configuration (***v***, ***h***) through the following Boltzmann distribution:

2 $$ p\left(\boldsymbol{v},\boldsymbol{h}\right)=\frac{1}{Z}{e}^{-E\left(\boldsymbol{v},\boldsymbol{h}\right)} $$

The partition function *Z* is given by summing over all possible pairs of configurations. The value of Z is generally unknown and computationally intractable, and is often the most challenging inference task for many prediction models. Since the visible vectors are our main concerns, we delve a step further into the probability of visible units *p*(***v***). We introduce a notation called free energy, which defined as

3 $$ f\left(\boldsymbol{v}\right)=- \log {\displaystyle {\sum}_{\boldsymbol{h}}{e}^{-E\left(\boldsymbol{v},\boldsymbol{h}\right)}} $$

And the energy function is only associated with visible units. With the definition of free energy, we can have the probability of visible vector as: $\boldsymbol{p}\left(\boldsymbol{v}\right)=\frac{1}{\boldsymbol{Z}}{\boldsymbol{e}}^{-\boldsymbol{f}\left(\boldsymbol{v}\right)}$. In particular, in binary RBM, the free energy turns into:

4 $$ \boldsymbol{f}\left(\boldsymbol{v}\right)=-{\displaystyle {\sum}_{\boldsymbol{i}}{\boldsymbol{b}}_{\boldsymbol{i}}{\boldsymbol{v}}_{\boldsymbol{i}} - {\displaystyle {\sum}_{\boldsymbol{j}}\mathbf{log}\left(\mathbf{1}+{\boldsymbol{e}}^{{\boldsymbol{c}}_{\boldsymbol{j}}+{\boldsymbol{W}}_{\boldsymbol{j}}\boldsymbol{v}}\right)}} $$

From the above equation, the energy (correspondingly, the probability) is determined by the weights and biases. Learning is conducted by performing stochastic gradient decent on the log likelihood of training data with respect to the individual parameter. Since the objective function in RBM is non-convex, the exact gradient is intractable. In this study, we used contrastive divergence (CD-k) [30] with *k* = 1 to achieve a reasonable approximation accuracy. In [31], the authors demonstrated that an RBM with sufficiently large number of hidden units can represent any distribution over binary vectors.

### Comparison between RBM and linear models

To demonstrate the representation performance of RBM in our experiments, it is worthwhile to compare our method to linear models. We now briefly turn to the discussion of two commonly used techniques, namely, PCA and linear discriminant analysis (LDA). Both of them are linear transformation methods and attempt to represent the data with lower dimensions. We refer the reader to references for more details [32, 33].

**Fig. 2:**
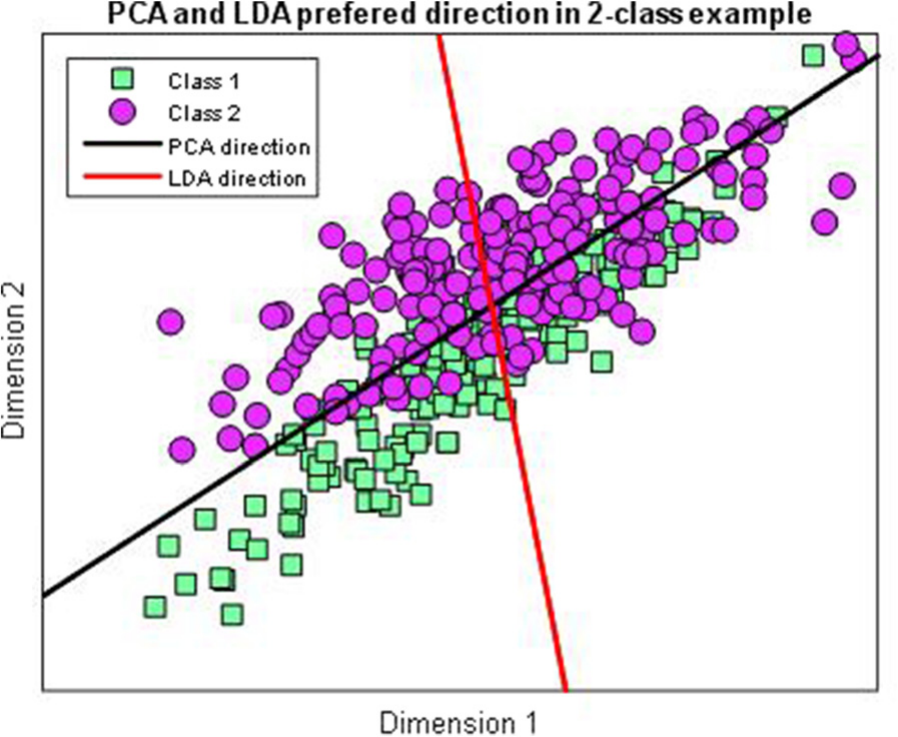
The main component direction found by PCA and LDA in 2-dimensional manner

PCA method finds a subspace, where basis vectors correspond to the maximum-variance directions in the original space. The principle behind is that a large variance usually has an important structure to consider. In practice, we keep only the largest *k* components to reduce the dimensions. When data was projected into this lower-dimensional space, we then fed them into some classifiers (we use support vector machine (SVM) in our experiment). In theory, however, PCA is not optimal for classification under some conditions, because it ignores the class discrimination. The discriminant dimensions could be simply discarded. A theoretically better method to find discriminant direction is LDA. It provides a linear boundary, which is generated by fitting class condition densities to the data. In a two-dimensional example shown in Fig. 2, PCA will prefer the direction, which is shown in black color, because it has the largest variance in the components directions while LDA (in red color) finds the direction that corresponds to the class discriminant direction. In this case, LDA should outperform PCA.

## Methods

### Original training data

In our study, we collected data from 7384 patients, which include 937,461 flowsheet entries in total. Based on our previous study [21] we selected 78 data items that were documented in continuous numerical values with sufficient frequencies. We synchronized the time series data using MATLAB.

### Classification using Discriminant RBM

We addressed the task of classification using RBM with two approaches. The first one is straightforward: we directly fed the hidden vectors into another classifier. Note that RBMs provide no guarantee that the generated hidden variables will ultimately be useful for the supervised task. In other words, if we are handling the task of 2-class classification and set the number of hidden units as 1, this hidden unit usually has no connection with our labels. The second more interesting approach is discriminant RBM, which utilizes the nature of a model to compute the probability.

**Fig. 3:**
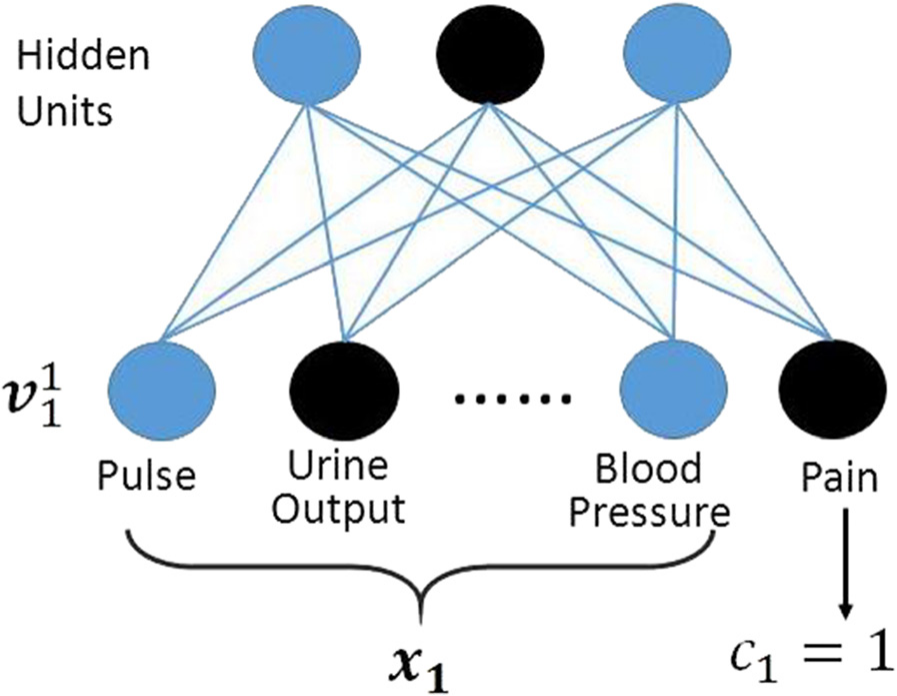
Pain label *c* is included in the visible layer. The *black* color denotes the state “on”, while the blue color denotes the state “off”

We assume that a test set *D*_*test*_ = {***v***_***i***_} = {(***x***_***i***_, *c*_*i*_)} consists of an input features vector ***x***_***i***_ and a target class *c*_*i*_ ∈ {0, 1} (see Fig. 3). The probabilities of two visible vector ***v***_1_^0^ = (***x***_1_, *c*_1_ = 0) and ***v***_1_^1^ = (***x***_1_, *c*_1_ = 1) can be directly computed from their free energies *f*(*v*_1_^0^) *and f*(*v*_1_^1^) through the equation (4). As shown in the equation (5), we can further obtain the probabilities *p*(***v***_1_^0^) *and p*(***v***_1_^1^) using the chain rule to cancel unknown constant *Z*.

5 $$ \frac{p\left({c}_1=0\Big|{x}_1\right)}{p\left({c}_1=1\Big|{x}_1\right)}=\frac{p\left({c}_1=0,\ {x}_1\right)}{p\left({c}_1=1,\ {x}_1\right)}=\frac{p\left({\boldsymbol{v}}_1^0\right)}{p\left({\boldsymbol{v}}_1^1\right)} $$

### Predicting pain state

In a supervised experiment, we would expect the number of labeled data points (i.e., training data) to be large. To this end, we only selected the features whose time interval was larger than the time interval of pain labels (i.e., documented pain). Four individuals (Table 1) with the number of recorded labels larger than 1000 were used in this experiment. None of the four patients had all 78 assessment items available. Number of available data items for the four patients varied from 22 to 28 (Table 1). We then randomly select approximately 20 % of the data as test data.

**Table 1:** Number of Data Items for Selected Individuals

Patient ID	Number of Data Items
*7137*	22
*4822*	28
*1245*	28
*6563*	24

In classification tasks it is necessary to perform pre-processing of the data before applying the algorithm. In our experiment, we converted the numerical data ***t*** into binary data ***x***. It is worth mentioning that the binary representation may be inappropriate in many real problems, although their interpretation (“normal” and “abnormal”) makes sense in our medical electronic data. Another important step is personalization, since the indication of a “normal” state varies among different individuals. We assumed the probability distribution of status measurement to be a Gaussian. We fit the numerical feature into this Gaussian probability distribution function, (*x*_*i*_ = 1) ~ ℕ(*t*_*i*_|*μ*, *σ*), where *p*(*x*_*i*_ = 1) represents the probability of current feature is abnormal. The mean and variance can be directly calculated from the samples. With this procedure, the resulting feature vectors are well-suited to the standard binary RBM. In this experiment, we converted non-binary probability into binary value by setting *threshold* = 0.5.

Since LDA allows only the *number of classes* – 1 dimension to be used, there is no parameter to be set in our 2-class task. For PCA, the size of the reduced dimension was selected as *k* = 4, which can cover most energy of the original data. In our experiment, we considered RBM as a non-parametric model and allowed the number of hidden variables to vary by the data [34]. As we have a trade-off to make: while a larger number of hidden units usually give a more powerful representation of distribution it also exaggerates the over-fitting problem. Therefore, the number of hidden units was adjusted between 15 and 30.

## Results

**Fig. 4:**
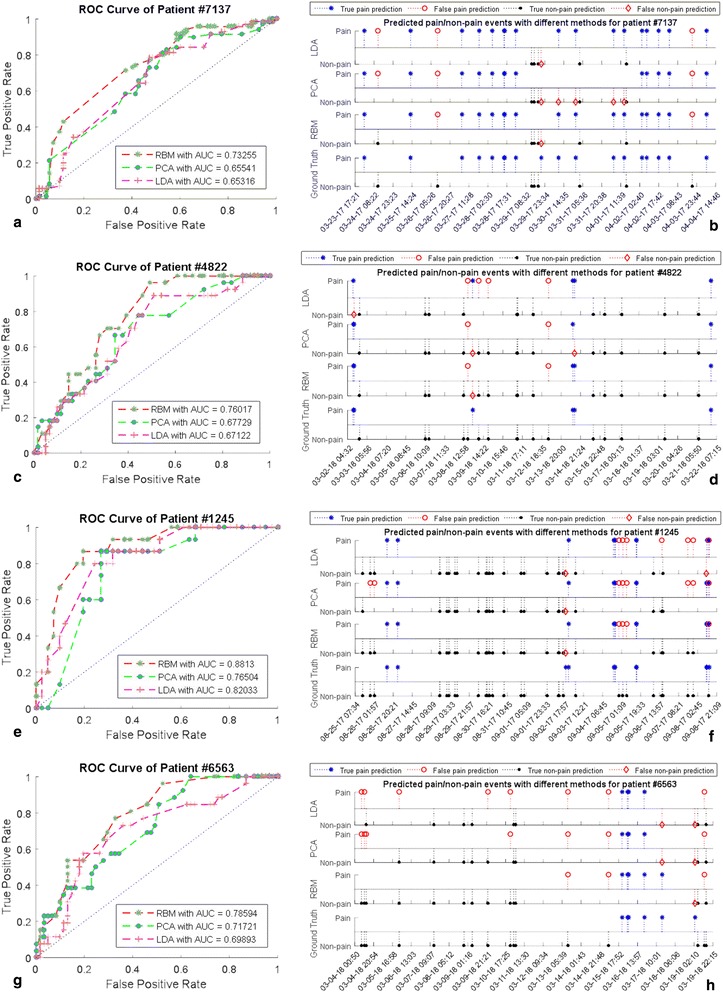
Comparison of RBM, LDA, and PCA in classification. (**a**), (**c**), (**e**) and (**g**) are the ROC curves and (**b**), (**d**), (**f**) and (**h**) are the predicted pain labels using their optimum threshold from patients (with IDs *7137*, *4822*, *1245*, and *6563*). Artificial timestamps are used for patient privacy

With regard to the classification performance, we first examined the Receiver Operating Characteristic (ROC) curves for each model and calculated the area under the curve (AUC) by varying the classification threshold (Fig. 4). Sensitivity, specificity and accuracy were used as the criteria of classification performance with the optimum boundary. The optimal point is defined as the minimum distance between the point (0,1) and any point on the ROC curve. Table 2 suggests that the performance of PCA and LDA is quite similar. Even though intuitive choice will prefer LDA to PCA, there is no guarantee that LDA will outperform PCA, especially when the size of training data is not sufficiently large. This observation is also reported in [35]. With carefully setting parameters, discriminant RBM can achieve better accuracy in all 4 experiments.

**Table 2:** Classification results using discriminant RBM, PCA with SVM, and LDA (Bold font denotes the best performance on certain metric of the 4 patients)

Patient ID	Model	AUC	Sensitivity	Specificity	Accuracy
*7137*	RBM	**0.73255**	0.7143	**0.6286**	**0.6714**
	PCA + SVM	0.65541	**0.8143**	0.4714	0.6429
	LDA	0.65316	0.8	0.4858	0.6429
*4822*	RBM	**0.76017**	**0.7037**	**0.6885**	**0.6932**
	PCA + SVM	0.67729	0.6667	0.6557	0.625
	LDA	0.67122	**0.7037**	0.5902	0.6591
*1245*	RBM	**0.8813**	**0.8667**	**0.8049**	**0.8214**
	PCA + SVM	0.76504	**0.8667**	0.7317	0.7679
	LDA	0.82033	0.8	0.7561	0.7679
*6563*	RBM	**0.78594**	**0.7692**	0.6721	**0.7011**
	PCA + SVM	0.71721	0.5769	**0.6885**	0.6552
	LDA	0.69893	0.7308	0.6393	0.6667

## Conclusions

Our experiments show that the RBM classification is competitive to the other methods such as LDA and SVM using PCA. The AUC was improved and the predicted pain labels using RBM outperformed the LDA and PCA using the optimum threshold, respectively. However, we notice that most results of AUC are still smaller than 0.8, which is only a fair level.

## Discussion

There are several limitations in our approach that might have contributed to this: (1) we have not given any consideration to the temporal data. Ignoring time will result in significant loss of information. We carried out the experiments by using the training data in the first half of time series, and made pain prediction with the test data, which corresponds to the second half of the time series, the detection rate of all methods turned out to be very poor. (2) Currently, we adopted the fundamental binary RBM. An obvious observation is that a binary representation may not be sufficient to represent states very well. For example, the value of blood pressure higher or lower than mean value may give totally different contributions to the pain response. In order to enrich the representation power, we will incorporate the softmax visible units to the model in our future work. (3) Most importantly, training of RBM expects the number of training data to be large. However, our experiment was done with a relatively small dataset. To this end, we will try to learn a model using a bigger dataset, and fine-tune the parameters only using the specific individual data in future work to minimize data loss.
